# Effect of Low Nanodiamond Concentrations and Polymerization Techniques on Physical Properties and Antifungal Activities of Denture Base Resin

**DOI:** 10.3390/polym13244331

**Published:** 2021-12-10

**Authors:** Shaimaa M. Fouda, Mohammed M. Gad, Passent Ellakany, Maram A. Al Ghamdi, Soban Q. Khan, Sultan Akhtar, Doaa M. Al Eraky, Fahad A. Al-Harbi

**Affiliations:** 1 Department of Substitutive Dental Sciences, College of Dentistry, Imam Abdulrahman Bin Faisal University, P.O. Box 1982, Dammam 31441, Saudi Arabia; mmjad@iau.edu.sa (M.M.G.); pellakany@iau.edu.sa (P.E.); maalghamdi@iau.edu.sa (M.A.A.G.); falharbi@iau.edu.sa (F.A.A.-H.); 2 Department of Dental Education, College of Dentistry, Imam Abdulrahman Bin Faisal University, P.O. Box 1982, Dammam 31441, Saudi Arabia; sqkhan@iau.edu.sa; 3 Department of Biophysics, Institute for Research and Medical Consultations, Imam Abdulrahman Bin Faisal University, P.O. Box 1982, Dammam 31441, Saudi Arabia; suakhtar@iau.edu.sa; 4 Department of Biomedical Dental Sciences, College of Dentistry, Imam Abdulrahman Bin Faisal University, P.O. Box 1982, Dammam 31441, Saudi Arabia; dmaleraky@iau.edu.sa

**Keywords:** acrylic resin, candidiasis, nanodiamonds, surface properties, polymerization

## Abstract

Background: Denture base resin has some drawbacks. This study investigated the impact of nanodiamonds (ND) and autoclave polymerization on the surface characteristics, translucency, and *Candida albicans* adherence in polymethyl methacrylate (PMMA) denture base resin after thermocycling. Methods: Heat-polymerized PMMA discs (15 × 2 mm) with a total sample size *n* = 160 were studied. Specimens were categorized into two main groups (*N* = 80): conventional water-bath-polymerized PMMA (CP/PMMA) and autoclave-polymerized PMMA (AP/PMMA). Each group was subdivided according to the ND concentration into four groups (*n* = 20): unmodified PMMA as a control, and 0.1%, 0.25%, and 0.5% ND–PMMA. Scanning electron microscopy (SEM) was used to inspect the morphology of the ND and the ND–PMMA mixtures before heat polymerization. The specimens were exposed to thermal cycling (5000 cycles at 5 and 55 °C), then surface roughness was measured with a non-contact optical interferometric profilometer, contact angle with an automated goniometer, and translucency using a spectrophotometer. Colony-forming units (CFU) were used to determine the adherence of *Candida albicans* cells to the specimens. ANOVA and Tukey post hoc tests for pairwise comparison were utilized for the statistical analysis (α = 0.05). Results: Surface roughness was significantly reduced with ND addition to CP/PMMA (*p* ˂ 0.001), while the reduction was not statistically significant in AP/PMMA (*p* = 0.831). The addition of ND significantly reduced the contact angle, translucency, and *Candida albicans* count of CP/PMMA and AP/PMMA (*p* ˂ 0.001). The incorporation of ND in conjunction with autoclave polymerization of PMMA showed significant reduction in all tested properties (surface roughness, contact angle and *Candida albicans* adherence) except translucency (*p* = 0.726). Conclusions: ND addition to PMMA and autoclave polymerization improved the surface properties with respect to antifungal activities, while the translucency was adversely affected.

## 1. Introduction

Polymethyl methacrylate (PMMA) is recommended in the manufacture of several dental appliances as it is characterized by being cost-effective, easy to process, and repairable, and has acceptable shade matching [1]. Nevertheless, the limited physical properties of PMMA make it less than ideal [2]. Moreover, the exposure of denture base resin to temperature changes adversely affects the resin properties [3]. To overcome these limitations, different methods have been suggested to improve the performance of PMMA, such as structural modifications by additives (chemicals, fillers, and nanofillers) and/or a different polymerization method [4].

A denture base material possessing hydrophilic properties and low surface roughness could reduce *Candida albicans* adhesion [5]. Coating the denture base was suggested to improve the surface properties of PMMA and reduce *Candida albicans* adhesion [6,7]. However, the durability of these coatings was doubtful [8]. The addition of antimicrobial agents to PMMA was also investigated, to increase denture resistance to microbial adhesion and consequently improve the oral health of denture wearers [8]. 

In addition, incorporation of reinforcing/antifungal agents and a different polymerization technique were used to enhance the mechanical characteristics of PMMA [4,9,10]. Recently, nanofillers have been used as a reinforcing agent in PMMA. Nanofillers have large active surfaces due to their small size; therefore, they could result in considerable changes in the properties of PMMA at low concentrations [8]. In addition, some nanofillers, such as nanodiamonds (ND), enhanced the antimicrobial activity of PMMA [11,12]. The antimicrobial activity of the ND might have arisen from oxygen-derived groups on their surface that interact with the components of bacterial cells, causing their death [13]. In addition, ND are biocompatible, have high strength, and are chemically stable [14]. They also link to PMMA through their reactive groups (NH_2_, OH) which enhance bonding with PMMA [15]. Al Harbi et al. [16] reported significant enhancement of the flexural strength and surface roughness of PMMA with the addition of 0.5% ND compared to higher concentrations (1% and 1.5% ND), while impact strength was reduced. The *Candida albicans* count was also decreased with ND addition, with the lowest count found at 1% for the ND/PMMA composite [12]. However, the observed color change of ND/PMMA, particularly at high ND concentrations, is considered a drawback [12]. 

PMMA is most commonly polymerized by being processed in a water bath, which is an uncomplicated conventional technique but requires long processing time [17]. However, other methods are also used for polymerization of PMMA, such as visible light, and autoclave and microwave methods, to speed up the polymerization of PMMA without causing any deterioration in the material composition and properties [9,10]. Polymerization of PMMA by autoclave is easier and can be done in less time than when using the conventional water-bath method [18]. Moreover, studies showed improved properties of the PMMA including improved flexural strength and surface hardness when the material was processed by autoclave, compared to the water-bath method [9,19,20]. Autoclave polymerization depends on the application of steam under high pressure and at higher temperatures. This procedure results in improvement of the PMMA properties by reducing the residual monomer content [21].

The effect of ND addition and autoclave polymerization on PMMA combined with thermal cycling has not been tested previously. The aim of the present study was to detect the effects of low levels of ND addition and autoclave polymerization on the surface properties and translucency of PMMA, as well as on *Candida albicans* adhesion after thermocycling. The first null hypothesis of the study states that addition of low amounts of ND would not change the tested properties of PMMA or the *Candida albicans* adhesion. The second null hypothesis of the study states that the properties of PMMA would be unchanged under the combined effect of ND addition and autoclave polymerization.

## 2. Materials and Methods

For the sample size calculation, the power was set at 80%, the level of significance was set at 5%, and the confidence interval was taken as 95%. Hence, the calculated sample size revealed that 160 disc-shaped specimens (15 × 2 mm) of heat-polymerized PMMA were required to carry out the study. The specimens were arranged in two main groups according to polymerization technique: conventional water-bath-polymerized PMMA (CP/PMMA) (*N* = 80) and autoclave-polymerized PMMA (AC/PMMA) (*N =* 80). Each group was subdivided according to the ND concentration into four groups (*n* = 20): unmodified as the control, and 0.1% ND–PMMA, 0.25% ND–PMMA, and 0.5% ND–PMMA. The materials used in the present study are shown in Table 1.

Heat treatment of the ND particles was performed at 450 °C for 120 min in air to release superficial functional groups [22]. An electronic balance (S-234; Denver Instrument GmbH, Göttingen, Germany) was employed to weigh the ND in concentrations of 0.1 wt.%, 0.25 wt.%, and 0.5 wt.% in acrylic resin powder. The ND particles were added to the acrylic resin powder and blended manually in a glass mortar and pestle with gentle hand pressure. Then, the samples mixed in an electric mixer at 400 rpm for 30 min at room temperature.

### Specimen Preparation and Processing 

Wax specimens were prepared using metal molds, then invested and flasked (61B Two Flask Compress; Handler Manufacturing, Westfild, NJ, USA). Mold spaces and all surfaces were painted with a separating medium after wax elimination. Heat-polymerized PMMA was prepared following the manufacturer’s instructions. Packing and polymerization were achieved using either the conventional water-bath method or by the autoclave polymerization method. Group 1 (CP) samples were conventionally heat-polymerized using the water-bath method, i.e., by inserting the flasks into a curing unit (KaVo Elektrotechnisches Werk GmbH, Leutkirch, Germany) for 8 h at 74 °C, and then raising the temperature to 100 °C for 60 min. Group 2 (AP) samples were autoclave polymerized by placing the flasks in an autoclave (Ritter M11 UltraClave; Midmark Corporation, Ohio, USA at 210 kPa (kilopascals) pressure and a temperature of 60 °C for 30 min, and then raising the temperature to 130 °C for 20 min [9,18].

After de-flasking, the excess resin was removed from the specimens with a tungsten carbide bur (HM251FX-040-HP; Meisinger, Centennial, CO, USA), followed by polishing with a mechanical polisher (MetaServ 250 grinder–polisher; Buehler, Lake Bluff, IL, USA) at 100 rpm for 120 s in wet conditions. The specimens were then placed in distilled water for 2 days at 37 °C.

Prior to examining the specimens, they were exposed to thermocycling (Thermocycler THE-1100, SD Mechatronik GmbH, Feldkirchen-Westerham, Germany) for 5000 cycles at 5 and 55 °C, with 5 s of transfer time and 30 s of dwell time, to simulate 6 months of actual use intraorally [23]. 

The surface roughness was evaluated with a non-contact optical interferometric profilometer with 0.01 mm resolution (Contour GT; Bruker Nano GmbH, Berlin, Germany). The specimens were placed horizontally below a standard camera at 20× magnification and the surface of each specimen was scanned across an area approximately 0.43 × 0.58 mm at five locations to obtain the average surface roughness value. Subsequently, the resulting images were visualized via a software package (Vision64; Bruker Nano, Coventry, UK) to detect pit features [24]. 

The contact angle was measured with an automated goniometer (DM-501; Kyowa Interface Science Co., Niiza, Japan) using the sessile drop method. After smoothly air-drying the specimen surface, a droplet of distilled water (2 μL) was placed on the surface using an auto-pipette. The average contact angle for each specimen was calculated by determining the tangent angle in relation to the water droplet surface at four distinct locations/specimen. FAMAS software (Kyowa Interface Science Co., Kyowa, Japan) was used to analyze the images [25]. 

The specimens’ reflectance values were detected using a spectrophotometer (Color-Eye 7000A spectrophotometer, X-Rite, Grand Rapids, MI, USA) after performing a calibration following the manufacturer’s recommendations. Specimens were kept against the port and supported by white or black backgrounds. A small-aperture viewing area was selected with dimensions of 10 × 7.5 mm. Four measurements were recorded/specimen to calculate the mean values of L*, a*, and b*. The Commission Internationale de l’Eclairage (CIE) system of L*, a*, and b* coordinates was used in the disc color measurements. CIE was used on the discs against each background. The translucency (TR) was analyzed utilizing the equation TR = [(L* white − L* black)^2^ + (a* white − a* black)^2^ + (b* white − b* black)^2^]^1/2^ [26]. 

The initial adhesion of *Candida albicans* to a specimen surface was performed to assess the first step of biofilm formation. The number of adhered cells was detected using colony-forming units (CFUs) as follows:

A *Candida albicans* reference strain (ATCC 10231) from a glycerol stock was streaked onto Sabouraud dextrose agar (SDA) plates two days prior to the assay, and plates were incubated at 30 °C for 48 h. A single colony was inoculated into 25 mL SDA broth medium to grow at 30 °C overnight and the yeast suspension was adjusted to 0.5 McFarland (approximately equivalent to 1 × 10^7^ cells/mL).

Transparent sterile 12-well microplates were used, and each specimen was sterilized using 70% isopropyl alcohol (IPA), then placed in a well with 1 mL of the fungal suspension and incubated at 37 °C for 90 min.

After incubation, 200 μL of phosphate buffer saline (PBS) was applied to the discs twice, to remove non-adhered fungal cells, then transferred to a new sterile Petri dish. To dislodge the adhered cells, 200 μL of PBS was added, scraping the surface with a pipette tip and homogenizing the solution by pipetting. Serial dilution was performed and 100 μL from the dilution tube was plated on SDA agar plates. The plates were incubated at 30 °C for 24–72 h, and colonies were counted twice, after 24 h and between 48 and 72 h, to ensure adequate growth and to distinguish colonies. The tests were conducted in three replicates. Positive and negative controls were carried out for each incubation time.

Statistical analysis was performed using SPSS v.23 software. The normality of the data for the tested samples was investigated using a Shapiro–Wilk test. Insignificant results from the test showed that the data were normally distributed; hence, parametric statistical tests were used for the data analysis. In the descriptive analysis, mean and standard deviations were computed. In the inferential statistics, one-way ANOVA was used to test the effect of variation in the concentration of nanodiamonds on the tested properties of heat-polymerized and autoclave-polymerized denture base material, followed by Tukey’s post hoc test for pairwise comparison. Additionally, a two-way ANOVA was employed to test the merged effect of the concentration levels and the polymerization method used. In the tests, *p*-values less than 0.05 were considered statistically significant.

## 3. Results

**Figure 1:**
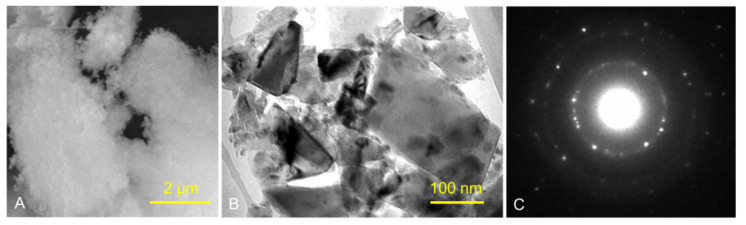
(**A**) SEM micrograph of ND powder, (**B**) TEM image of ND powder, and (**C**) corresponding selected-area electron diffraction (SAED) pattern of ND crystalline particles.

**Figure 2:**
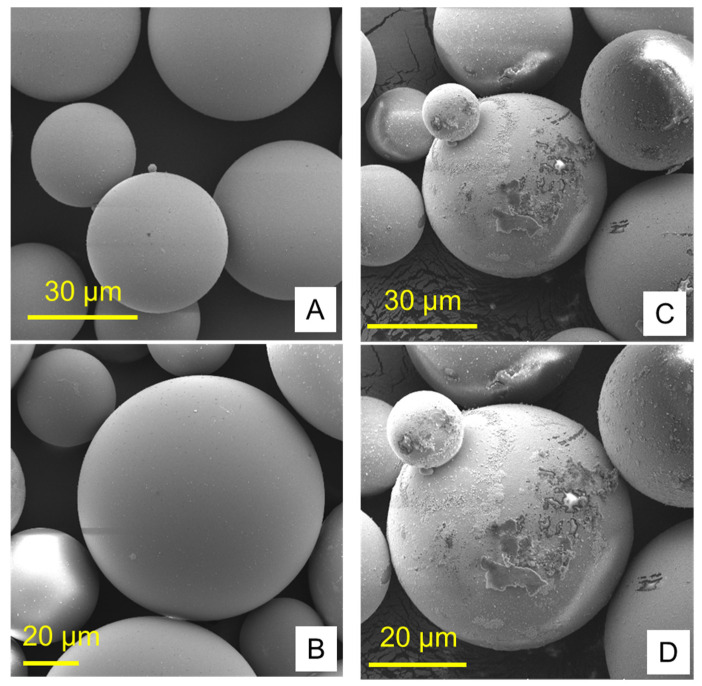
Scanning electron micrographs of (**A**,**B**) pure PMMA and (**C**,**D**) PMMA/ND mixture at two magnifications.

Figure 1 and Figure 2 show the structures of pure ND and PMMA, and the distribution of ND in the PMMA (ND/PMMA mixture) under scanning electron microscopy (SEM). In addition, the detailed features and the configuration of ND particles were visualized at high resolution in the ND powder by transmission electron microscopy (TEM). 

Table 2 shows the mean and standard deviation values of the surface roughness, contact angle, translucency, and *Candida albicans* count. The surface roughness, contact angle, and *Candida albicans* count of conventionally polymerized PMMA (CP) were found to be highest in the control group, while the lowest values of *Candida albicans* and contact angle were found at 0.5% ND–PMMA. The lowest surface roughness value was found at 0.25% ND–PMMA. The translucency of conventionally polymerized and autoclave-polymerized PMMA was found to be highest in the control and lowest at 0.5% ND–PMMA (Table 2).

In the autoclave-polymerized group (AP), the average surface roughness was highest for 0% and 0.25% ND–PMMA and lowest for 0.1% ND–PMMA. Similarly, the maximum value for *Candida albicans* was obtained at 0.1% ND–PMMA and the minimum value at 0.5% ND–PMMA. The contact angle value was at a maximum in the control group and its minimum value was found at 0.5% ND–PMMA (Table 2). Furthermore, the one-way ANOVA results revealed a significant association (*p* ˂ 0.001) between concentration levels of nanodiamond and the tested properties in conventionally polymerized PMMA. However, in the autoclave group, concentration levels were insignificantly associated (*p* = 0.831) with surface roughness but significantly associated (*p* ˂ 0.001) with contact angle, translucency, and *Candida albicans* count (Table S1).

**Figure 3:**
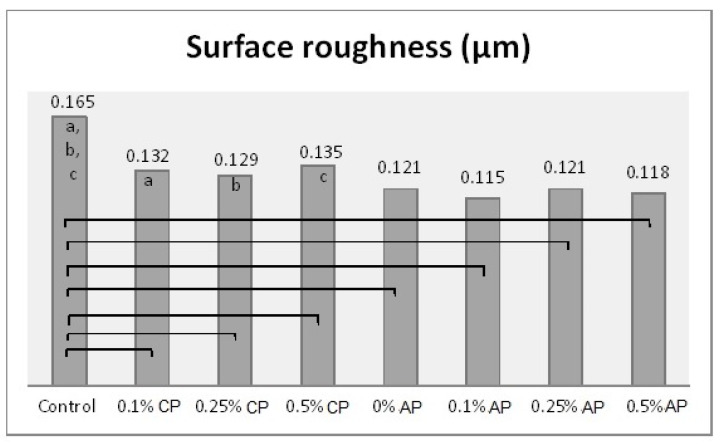
Mean of surface roughness (µm) and significances between groups for ND concentrations and polymerization techniques. Same lower-case letters a, b, c show significant differences in mean within CP group. Lines show the significant differences between all groups comparing polymerization techniques.

**Figure 4:**
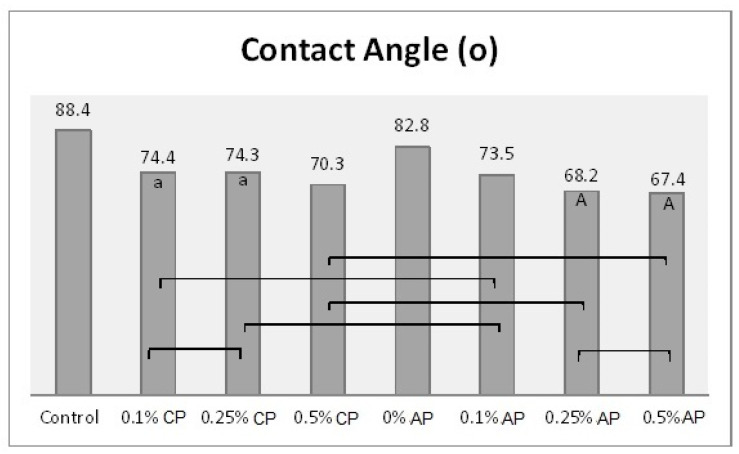
Mean of contact angle (^o^) and significances between groups for ND concentrations and polymerization techniques. Same lower-case letters a show insignificant differences in means within CP group. Same capital letters A show insignificant differences in means within AP group. Lines show the insignificant differences between all groups comparing polymerization techniques.

**Figure 5:**
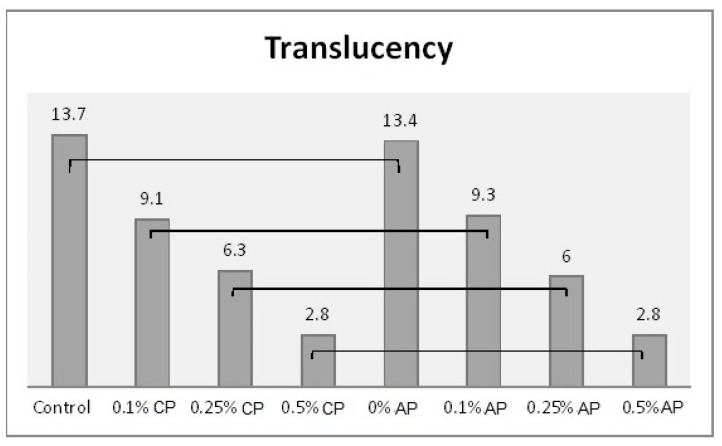
Mean of translucency and significances between groups for ND concentrations and polymerization techniques. Lines show statistically insignificant differences in means comparing polymerization techniques.

**Figure 6:**
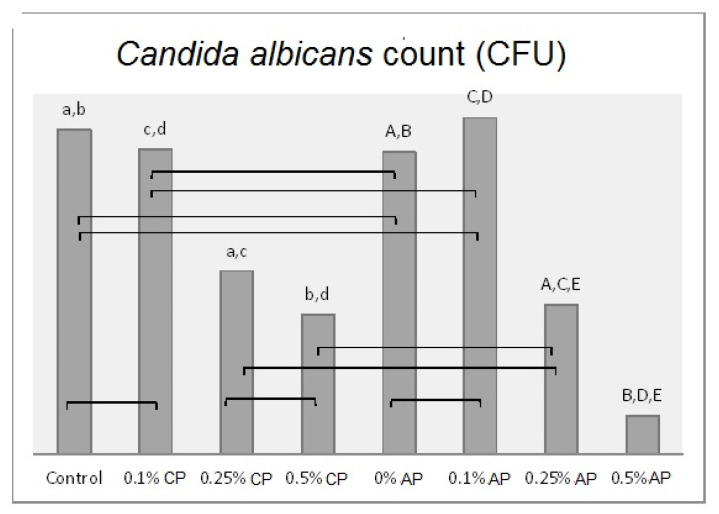
Mean of *Candida albicans* (CFU/mL) count and significances between groups for ND concentrations and polymerization techniques. Same lower-case letters a, b, c, d show significant differences between the means in CP group. Same upper-case letters A, B, C, D, E show significant differences between the means in AP group. Lines show the insignificant differences comparing polymerization techniques.

One-way ANOVA results were obtained after combining the groups (CP and AP) together. Hence, there were eight different concentrations (as factors for ANOVA) which were analyzed with the tested properties. The effect of concentration levels appeared to be statistically significant (*p* *˂* 0.001) over all the tested properties (Table S2). The Tukey post hoc test was applied for pairwise comparison. Figure 3, Figure 4, Figure 5 and Figure 6 present the post hoc test results. 

**Figure 7:**
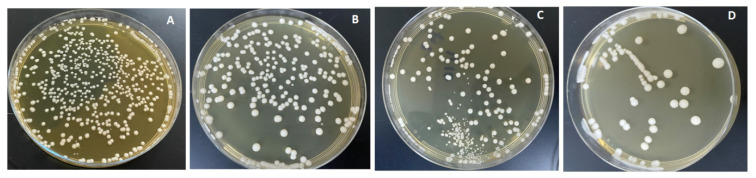
Cultures of *Candida albicans* colonies in CP/PMMA in (**A**) pure PMMA, (**B**) 0.1%, (**C**) 0.25%, and (**D**) 0.5% ND.

**Figure 8:**
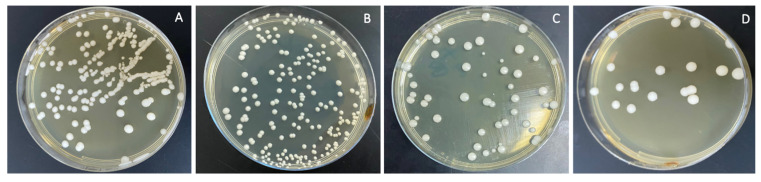
Cultures of *Candida albicans* colonies in AP/PMMA in (**A**) pure PMMA, (**B**) 0.1%, (**C**) 0.25%, and (**D**) 0.5% ND.

**Figure 9:**
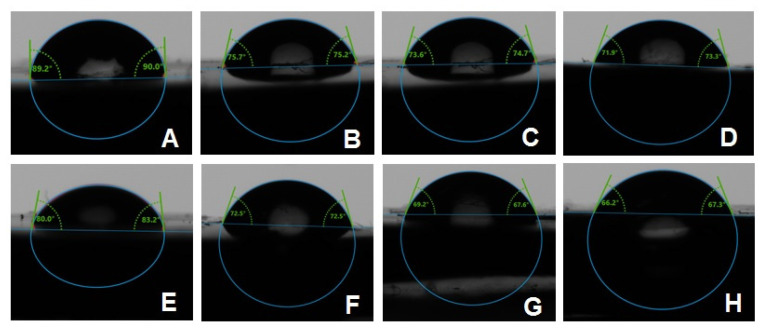
Representative contact angle images of CP groups: (**A**) unmodified, (**B**) 0.1% ND, (**C**) 0.25% ND, (**D**) 0.5% ND; and AP groups: (**E**) unmodified, (**F**) 0.1% ND, (**G**) 0.25% ND, (**H**) 0.5% ND.

The combined effects of ND concentration and polymerization method showed **a** significant effect on all tested properties except translucency, as revealed by the two-way ANOVA test (Table 3). Figure 7 and Figure 8A–D show Candida albicans colonies in CP and AP for pure PMMA and 0.1, 0.25, and 0.5% ND, respectively. The fewest Candida albicans colonies can be observed in Figure 8D, while Figure 7A shows the highest number of colonies. Figure 9 shows the contact angles in CP and AP groups.

## 4. Discussion

The first null hypothesis was rejected because the addition of ND improved the surface properties of PMMA and also reduced the adhesion of *Candida albicans*, but translucency was adversely affected. The second null hypothesis was also rejected. The addition of ND and autoclave polymerization resulted in a reduction in surface roughness, contact angle, *Candida albicans* adherence to PMMA, and translucency.

It is essential to investigate the properties of PMMA under conditions mimicking actual use intraorally. Therefore, the specimens in this study were exposed to thermal cycling simulating 6 months of actual use, in order to imitate the thermal changes occurring intraorally as a result of food and drinks [23]. This adds to the credibility of the study; however, longer exposure to thermal changes is required to assess the long-term effect of thermal cycling on ND-reinforced PMMA. 

Denture base material must have a smooth surface to reduce microbial adhesion [27]. The results showed a significant reduction in surface roughness as a result of ND addition in conventionally polymerized PMMA (CP/PMMA). Previous studies [12,16] found a reduction in surface roughness with ND addition to PMMA at 0.5%, which agrees with the findings of the present study. The addition of nanoparticles fills the pores of the polymer, thus reducing the surface roughness. However, at high concentrations agglomeration of the nanoparticles could cause an opposite effect [16]. Although ND concentration did not significantly change the surface roughness for autoclave-polymerized PMMA (AP/PMMA) subgroups, the surface roughness of AP/PMMA was inferior to that of CP/PMMA. Moreover, the combined effect of ND addition and autoclave polymerization resulted in a significant reduction in the surface roughness of PMMA in the present study. However, Gad et al. [9] reported an insignificant difference in surface roughness between conventionally polymerized and autoclave-polymerized PMMA. The difference in results may be due to the addition of ND to autoclave-polymerized PMMA in the present study and/or the exposure of specimens to thermal cycling.

The findings of the present study showed a significant reduction in contact angle of ND/PMMA in both groups with different polymerization methods. Moreover, autoclave polymerization was associated with lower contact angle values compared to conventional polymerization. This decrease may be attributed to the impact of added nanoparticles on the surface characteristics of PMMA and the reduction of surface tension [25]. Similarly, previous studies found a reduction in the contact angle of PMMA after the addition of various nanoparticles, including silicon oxide, titanium oxide, and zirconium oxide nanoparticles [25,28,29]. However, a previous study showed an insignificant difference in contact angle between pure PMMA and PMMA reinforced with ND at 0.5, 1, and 1.5% [12]. The variation in results might be due to different methods employed in the polymerization, different ND concentrations, or the exposure of the specimens to thermal cycling. 

Translucency provides a natural appearance to denture base materials by allowing the passage of light through denture resin, thus reflecting the shade of normal healthy soft tissue. Variations in the denture resin composition and surface roughness reduce light refraction, which in consequence reduces translucency [26]. The findings of the current study showed low translucency for the 0.5% ND conventionally polymerized and autoclave-polymerized PMMA. This is in agreement with previous studies which reported a reduction in the translucency of PMMA denture base resins incorporating fillers or nanofillers such as nano-ZrO_2_ particles [26], zirconium oxide, silicon oxide and aluminum oxides, particularly at high filler concentrations [30]. The decrease in translucency might be related to the presence of different filler types possessing dissimilar optical properties [30]. In addition, the increase in filler concentration could result in the formation of clusters that prevent light diffusion through the resin. Moreover, ND possess a higher refractive index (2.11) than PMMA (1.48) [26,31]. Thus, the difference in the refractive indices of ND and PMMA resin explains the reduction in translucency of PMMA denture resin and its opaquer appearance [32]. In a recent study, Gad et al. [33] demonstrated a reduction in PMMA translucency with the addition of different nanofillers at concentrations between 0.5–2.5%, including ND, which showed the lowest translucency amongst the tested nanoparticles. This could be due to the gray color of ND and its sheet-like morphology, which might have reduced light transmission [33]. 

Significant reduction of *Candida albicans* adherence with ND addition was reported in this study. In addition, autoclave polymerization with ND addition significantly reduced *Candida albicans* adhesion. The lowest count of *Candida albicans* was found with the addition of 0.5% ND in AC/PMMA followed by CP/PMMA. Previous studies reported a reduction in *Candida albicans* adhesion with the addition of ND to heat-polymerized and autopolymerized PMMA, which agrees with the present findings [12,34]. The reason could be the antimicrobial effect of ND, which has been reported in several studies [13,14,35,36]. The antimicrobial effect of ND might result from interaction between its surface oxygen-derived groups and bacterial cells or might be due to its antiadhesive characteristics [13,37,38]. In addition, the impact of ND on the surface characteristics of PMMA was reported in the present study, including the reduction in the surface roughness and contact angle of conventionally polymerized or autoclave-polymerized ND/PMMA. The decrease in surface roughness reduces the area available for microbial adhesion [5]. Therefore, a reduction in surface roughness could reduce colonization of *Candida albicans*, the causative microorganism of denture stomatitis. Moreover, some studies noted a link between low contact angle and decreased fungal adhesion [39,40]. It was mentioned that hydrophilic surfaces reduce fungal adhesion compared to hydrophobic surfaces, which form strong hydrophobic bonds with microbes [41]. In line with the results of the present study, the smallest *Candida albicans* count was found in the AP/PMMA group with 0.5% ND, which had the lowest contact angle value among all the tested groups. 

Clinically, the oral health of denture wearers could be enhanced by increasing denture resistance to *Candida albicans* adhesion and improving the surface properties through ND addition and autoclave polymerization, even after exposure to thermal stress. Therefore, low levels of ND addition to PMMA denture base materials in combination with autoclave polymerization could be recommended for denture base fabrication. 

Although the specimens in this study were exposed to thermal stress before testing, they were not subjected to all intraoral conditions, such as variation in pH values, exposure to saliva, food, and beverages, and denture cleaning routines. Therefore, in vivo studies are needed to test the effects of these factors and the durability of the ND effect on PMMA. It is also recommended that ND be added to PMMA at the fitting surface or in non-esthetic areas, to avoid the disadvantages of the significant reduction of PMMA’s translucency, as was suggested in a previous study [42].

## 5. Conclusions

The surface roughness, contact angle, and *Candida albicans* adherence were reduced by ND addition and autoclave polymerization. Translucency was adversely affected by ND addition but showed an insignificant difference with regard to the polymerization method.

## Figures and Tables

**Table 1:** Materials used.

Materials	Brand/Supplier
Heat-polymerized PMMA	Major base 20, Major Prodotti Dentari Spa, Moncalieri, Italy
Nanodiamond	Shanghai Richem International Co., Ltd., Shanghai, China
Base plate wax	Set-up Wax; Cavex, Haarlem, The Netherlands
Dental stone	Fujirock EP; GC, Leuven, Belgium
Separating medium	Isol Major; Major Prodotti Dentari Spa, Moncalieri, Italy

**Table 2:** Average and standard deviation values of tested properties.

Groups	Concentration	Surface Roughness (µm)	*C*. *albicans* (cfu/mL)	Contact Angle (°)	Translucency
CP	Control	0.165 (0.02)	16,220 (4973.4)	88.4 (1.6)	13.7 (1.1)
	0.1%	0.132 (0.01)	15,240 (5474.2)	74.4 (1.7)	9.1 (0.7)
	0.25%	0.129 (0.02)	9160 (1487.9)	74.3 (1.1)	6.3 (0.6)
	0.5%	0.135 (0.02)	6980 (831.1)	70.3 (1.4)	2.8 (0.4)
AP	0%	0.121 (0.02)	15,100 (3177.7)	82.8 (3.1)	13.4 (0.7)
	0.1%	0.115 (0.01)	16,820 (4683.6)	73.5 (2.3)	9.3 (0.72)
	0.25%	0.121 (0.01)	7460 (1780.9)	68.2 (3.3)	6.0 (0.73)
	0.5%	0.118 (0.02)	1930 (583.2)	67.4 (2.7)	2.8 (0.39)

**Table 3:** Combined effect of ND concentration and polymerization methods using two-way ANOVA.

Property	Source	Type III Sum of Squares	*df*	Mean Square	*F*	*p*
Surface roughness	concentration	0.005	3	0.002	5.179	0.003 *
	type	0.009	1	0.009	29.017	<0.0001 *
	concentration * type	0.004	3	0.001	3.813	0.014 *
	Error	0.023	72	0.000		
	Total	1.382	80			
*C. albicans*	concentration	1,940,757,375.000	3	646,919,125.000	55.557	<0.0001 *
	type	49,455,125.000	1	49,455,125.000	4.247	0.043 *
	concentration * type	111,261,375.000	3	37,087,125.000	3.185	0.029 *
	Error	838,381,000.000	72	11,644,180.556		
	Total	12,821,090,000.000	80			
Contact Angle	concentration	3309.149	3	1103.050	210.601	<0.0001 *
	type	299.538	1	299.538	57.190	<0.0001 *
	concentration * type	89.465	3	29.822	5.694	0.001 *
	Error	377.110	72	5.238		
	Total	453,055.840	80			
Translucency	concentration	1259.733	3	419.911	875.552	<0.0001 *
	type	0.277	1	0.277	0.578	0.450
	concentration * type	0.631	3	0.210	0.439	0.726
	Error	34.531	72	0.480		
	Total	6323.585	80			

* Statistically significant at 0.05 level.

## Data Availability

Data are available upon request from the corresponding author.

## Supplementary Materials

The following are available online at https://www.mdpi.com/article/10.3390/polym13244331/s1, Table S1: Effect of different ND concentration levels on tested properties of conventional and autoclave polymerized PMMA. Table S2: Effect of different ND concentration levels on tested properties (after combining both groups).
