# Supplementary material for: Effect of Low Nanodiamond Concentrations and Polymerization Techniques on Physical Properties and Antifungal Activities of Denture Base Resin
Source: Polymers (Basel). 2021 Dec 10;13(24):4331. doi: 10.3390/polym13244331 (PMC8708888; doi:10.3390/polym13244331)
Supplement: Supplementary file 1 [file polymers-13-04331-s001.zip › polymers-1469783-supplementary.pdf]

Table S1: Effect of different ND concentration levels on tested properties of conventional and autoclave polymerized PMMA.

Material	Property	Groups	Sum of Square	df	Mean Square	F-value	P
CP	Surface Roughness	Between groups	.008	3	.003	7.856	<0.0001*
		Within groups	.013	36	.000		
		Total	.021	39			
	C. albicans	Between groups	615320000.0	3	205106666.7	14.242	<0.0001*
		Within groups	518460000.0	36	14401666.7		
		Total	1133780000.0	39			
	Contact Angle	Between groups	1897.778	3	632.593	288.482	<0.0001*
		Within groups	78.942	36	2.193		
		Total	1976.720	39			
	Translucency	Between groups	635.0	3	211.679	384.4	<0.0001*
		Within groups	19.82	36	0.551		
		Total	654.8	39			
**AP**	Surface Roughness	Between groups	.000	3	.000	.291	0.831
		Within groups	.010	36	.000		
		Total	.010	39			
	C. albicans	Between groups	1436698750.0	3	478899583.333	53.890	<0.0001*
		Within groups	319921000.0	36	8886694.444		
		Total	1756619750.0	39			
	Contact Angle	Between groups	1500.836	3	500.279	60.402	<0.0001*
		Within groups	298.168	36	8.282		
		Total	1799.004	39			
	Translucency	Between groups	625.327	3	208.442	510.225	<0.0001*
		Within groups	14.707	36	0.409		
		Total	640.034	39			

*Statistically at 0.05 level of significance

Table S2: Effect of different ND concentration levels on tested properties (after combining both groups)

**Property**	**Groups**	**Sum of Square**	***df***	**Mean Square**	***F***	***P***
**Surface Roughness**	Between groups	.018	7	.003	7.999	<0.0001*
	Within groups	.023	72	.000		
	Total	.041	79			
C. albicans	Between groups	2101473875.0	7	300210553.571	25.782	<0.0001*
	Within groups	838381000.0	72	11644180.556		
	Total	2939854875.0	79			
**Contact Angle**	Between groups	3698.152	7	528.307	100.867	<0.0001*
	Within groups	377.110	72	5.238		
	Total	4075.262	79			
**Translucency**	Between groups	1260.641	7	180.092	375.507	<0.0001*
	Within groups	34.531	72	.480		
	Total	1295.172	79			

*Statistically at 0.05 level of significance
